# Remote cortical atrophy and language outcomes after chronic left subcortical stroke with aphasia

**DOI:** 10.3389/fnins.2022.853169

**Published:** 2022-08-03

**Authors:** Huijia Tang, Shuhan Fan, Xingyang Niu, Zhuhao Li, Peiyi Xiao, Jinsheng Zeng, Shihui Xing

**Affiliations:** ^1^Department of Neurology and Stroke Center, Guangdong Provincial Key Laboratory of Diagnosis and Treatment of Major Neurological Diseases, National Key Clinical Department and Key Discipline of Neurology, The First Affiliated Hospital, Sun Yat-sen University, Guangzhou, China; ^2^Department of Radiology, The First Affiliated Hospital, Sun Yat-sen University, Guangzhou, China

**Keywords:** aphasia, cortical thickness, diffusion tensor imaging, subcortical stroke, outcomes

## Abstract

**Objective:**

Subcortical stroke can cause a variety of language deficits. However, the neural mechanisms underlying subcortical aphasia after stroke remain incompletely elucidated. We aimed to determine the effects of distant cortical structures on aphasia outcomes and examine the correlation of cortical thickness measures with connecting tracts integrity after chronic left subcortical stroke.

**Methods:**

Thirty-two patients and 30 healthy control subjects underwent MRI scanning and language assessment with the Western Aphasia Battery-Revised (WAB-R) subtests. Among patients, the cortical thickness in brain regions that related to language performance were assessed by the FreeSurfer software. Fiber tracts connecting the identified cortical regions to stroke lesions were reconstructed to determine its correlations with the cortical thickness measures across individual patient.

**Results:**

Cortical thickness in different parts of the left fronto-temporo-parietal (FTP) regions were positively related to auditory-verbal comprehension, spontaneous speech and naming/word finding abilities when controlling for key demographic variables and lesion size. Cortical thickness decline in the identified cortical regions was positively correlated with integrity loss of fiber tracts connected to stroke lesions. Additionally, no significant difference in cortical thickness was found across the left hemisphere between the subgroup of patients with hypoperfusion (HP) and those without HP at stroke onset.

**Conclusions:**

These findings suggest that remote cortical atrophy independently predicts language outcomes in patients with chronic left subcortical stroke and aphasia and that cortical thinning in these regions might relate to integrity loss of fiber tracts connected to stroke lesions.

## Introduction

Aphasia is observed in about one third of patients with ischemic or hemorrhagic stroke. Empirically, aphasia is attributed to lesions in cortical regions such as Broca’s and Wernicke’s areas after stroke. In a follow-up computed tomography investigation, stroke lesion has been demonstrated to predict the outcomes of post-stroke aphasia at a modest degree (Worrall et al., 2001). In contrast, language deficits have been reported in patients with subcortical lesions in the internal capsule, putamen, thalamus, basal ganglia, and periventricular white matter (Naeser et al., 1982; Kuljic-Obradovic, 2003; Dronkers et al., 2017). At present, the underlying mechanisms of subcortical aphasia remain incompletely known.

Accumulating evidence has suggested subcortical aphasia to be resultant of cortical hypoperfusion (HP) in brain regions that support language function. Previous studies have showed that the degree of cortical HP is associated with language outcomes in patients with acute subcortical stroke (Olsen et al., 1986; Perani et al., 1987; Okuda et al., 1994; Hillis et al., 2002). The severity of subcortical aphasia has been found to relate to the degree of cortical HP (Choi et al., 2007). In contrast, language deficits can be recovered when cortical perfusion is regained (Vallar et al., 1988). Even in the chronic stage of subcortical stroke, patients might have language deficits that relate to persistent cortical HP (Peñaloza et al., 2014). However, a recent study has showed that cortical HP was not observed in most patients with left thalamic infarction and aphasia (Sebastian et al., 2014). These findings imply that potential alternative mechanisms might be involved in subcortical aphasia.

It has been shown that subcortical stroke can lead to structural changes in remote cortical regions. The role of remote cortical changes in functional outcomes is controversial. Motor functional recovery is related to gray matter volumes of cortical regions (Dang et al., 2013), or cortical thickness measures (Lotan et al., 2019) following subcortical stroke. Other studies have showed remote cortical atrophy that determined by connectivity to the primary lesion was not related to motor deficits after subcortical stroke (Cheng et al., 2015; Liu et al., 2020). With respect to aphasia, secondary cortical degeneration may result from impairment of white matter tracts that are crucial for reorganization after stroke (Duering et al., 2015). Integrity of arcuate fasciculus and uncinate fasciculus can predict the severity and recovery of subcortical aphasia (Noh et al., 2021; Zhang et al., 2021). While great efforts have been devoted to understanding the neural basis for subcortical aphasia, the effects of subcortical stroke on cortical thickness and language outcomes remain incompletely investigated. As the left fronto-temporo-parietal (FTP) cortex is commonly implicated in language processes. It might be expected that cortical regions beyond the subcortical lesion may undergo atrophy due to disconnection and have a negative impact on language outcomes following subcortical stroke in the left hemisphere.

To text this hypothesis, we aimed to determine the potential correlates of cortical thickness with language outcomes in patients with chronic left subcortical stroke and aphasia. By combining tractography with cortical thickness measures, we further explored the possible relationships between cortical thickness changes and integrity of fiber tracts connected to subcortical lesions. We hypothesized that subcortical stroke induced cortical thickness changes in connecting brain regions and related to language outcomes at the chronic stage of subcortical stroke.

## Materials and methods

### Participants

Thirty-two patients with chronic left subcortical stroke (ischemic or hemorrhagic) and history of aphasia were recruited with the following inclusion criteria: (i) Native Chinese speakers; (ii) over 6 months after stroke; (iii) unilateral subcortical lesion; (iv) ability to perform tests; and (v) absence of prior diagnosis of neurological disease. Based on medical records, all patients had aphasia at the time of stroke and received varied types of speech–language rehabilitation. Thirty age-matched healthy subjects with no history of neurological disease were recruited in the study.

The study was approved by the Institutional Review Board of the First Affiliated Hospital of Sun Yat-sen University, and written informed consent was obtained from all participants before enrollment in the study.

### Language assessment

Language performance of all participants was assessed using the Western Aphasia Battery-Revised (WAB-R) (Shewan and Kertesz, 1980), which was translated into Chinese version. The language assessment was administrated and scored by two neurologists (S.H.F and H.J.T). The WAB-R includes separate subtests that provide composite scores for spontaneous speech, repetition, naming/word finding, and auditory-verbal comprehension. The total of these composite scores is the aphasia quotient, a measure of overall aphasia severity ranging from 0 to 100.

### Image acquisition

Image data were acquired on a Siemens 3T Trio scanner with a 12-channel coil, and three-dimensional T1-weighted structural images were acquired using the magnetization-prepared rapid gradient-echo imaging sequence: TR (relaxation time) = 1,900 ms; TE (echo time) = 2.56 ms; flip angle = 9°; field of view (FOV) = 250 mm × 250 mm; 192 contiguous 1-mm sagittal slices; voxel size = 1 mm × 1 mm × 1 mm). Diffusion-weighted images were acquired using the single-shot echo-planar imaging sequence: TR = 8,700 ms; TE = 90 ms; flip angle = 90°; FOV = 240 mm × 240 mm; 64 3-mm sagittal slices; voxel size = 2 mm × 2 mm × 3 mm; and 64 diffusion volumes weighted with *b*_max_ = 1,100 s/mm^2^ and one volume with no diffusion gradient (*b*_0_ = 0 s/mm^2^).

### Data preprocessing

#### Structural data preprocessing

**FIGURE 1:**
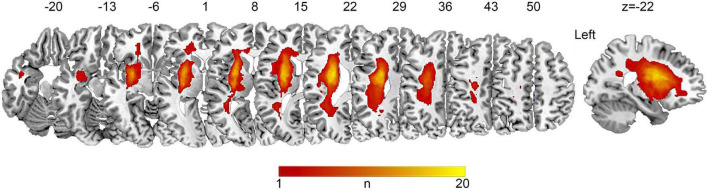
Lesion overlap map of patients with chronic subcortical stroke. Lesions from 32 patients were normalized to the MNI space. The *n*-value denotes the number of patients with a lesion in each voxel (maximum 20 out of 32).

Subcortical stroke lesions were segmented on the T1-weighted images in native space using MRIcron software^1^ and independently confirmed by two neurologists (S.H.F and S.H.X). The raw lesion maps were then normalized to the Montreal Neurological Institute (MNI) space so as to allow direct comparisons across individuals, using the enantiomorphic approach (Nachev et al., 2008) with minor modifications by using the unified segmentation approach in SPM12 with CAT12.^2^ The lesion overlap map is shown in Figure 1.

Support vector regression-based lesion-symptom mapping (SVR-LSM), a multivariate lesion-symptom mapping approach, was employed to examine critical subcortical areas in the left hemisphere in which damage relates to language deficits as previously described (DeMarco and Turkeltaub, 2018). SVR-LSM analyses were performed for spontaneous speech, naming/word finding, repetition and auditory-verbal comprehension, respectively. Only voxels damaged in at least 10% of patients were included in the analysis. Probabilistic maps created using 10,000 permutations of the behavioral scores were thresholded at a voxel-level of *P* < 0.005 and cluster-level of *P* < 0.05.

The structural MRI data were preprocessed using the automated surface-based analysis package FreeSurfer (version 7.1.1^3^) with standard procedures and parameters. Cortical thickness measures by FreeSurfer software have been confirmed by histological and manual measurements (Fischl et al., 2004). The semiautomatic processes included motion correction, intensity normalization, non-linear registration, white and gray matter segmentation, and surface mesh representation of the cortex. All images were visually inspected, and cortical segmentations were manually corrected if necessary. Cortical thickness maps were registered to the FreeSurfer surface template (fsaverage) and smoothed with a full-width half maximum Gaussian kernel of 15 mm.

#### Diffusion data preprocessing

Diffusion-weighted images were preprocessed following the standard protocols using the FMRIB software library (FSL^4^). The main procedure included the following steps: skull removal with the brain extraction tool, correction for head motion and eddy current distortions using FMRIB’s Diffusion Toolbox, building diffusion tensor models, and calculation of fractional anisotropy (FA) maps using the diffusion tensor imaging fractional intensity threshold (DTIFIT) tools.

#### White matter connectivity reconstruction

For fiber tracking, the cortical regions that exhibited association with language outcomes were transformed back to the native DWI space, and the transformed cortical regions and lesion mask in native space were defined as paired regions of interest (ROIs). To reconstruct the fiber tracts in the contralesional hemisphere, we defined homologous ROIs in the contralateral hemisphere based on the mirrored stroke lesion and cortical regions for each patient. The representative ROIs in subject native space are shown in Supplementary Figure 1. Connections between the lesion and cortical ROIs or their mirrored homologs were reconstructed using the probabilistic tracking of crossing fibers (probtrackx) and Bayesian estimation of diffusion parameters (bedpostx) algorithms implemented in FSL as previously described (Smith et al., 2004; Behrens et al., 2007). Briefly, fiber tracking was conducted using subcortical lesion as seed and cortical regions as target in native space of individual patient. From each mask voxel, 5,000 streamline samples with a step length of 0.5 mm and a curvature threshold of 0.2 were generated to map the probabilistic connection pattern. The tract density map was obtained by dividing by the total number of streamline samples. Fiber tracking was performed in both directions from seed to target and backward, and the connection maps were averaged. The final tract density map was obtained by dividing by the total number of streamlined samples, and then thresholded at 1% to exclude spurious connections as suggested in the previous studies (Duering et al., 2012; Cheng et al., 2015, 2020; Xing et al., 2018). Thereafter, FA values of reconstructed connections were extracted for individual patients. For visual inspection, the resultant tracts were normalized to the MNI space to show the connections within each voxel of the tract across patients with the non-linear parameters using the FNIRT tool in FSL.

### Statistical analysis

To determine cortical thickness association with language measures, cortical thickness maps were entered into separate multiple regression analyses using spontaneous speech, naming/word finding, repetition, and auditory-verbal comprehension as explanatory variables controlling for key demographic factors and lesion size as confounding variables. Multiple comparisons were corrected at a voxel-wise threshold of *p* < 0.005 and a cluster-wise level of *p* < 0.05, as empirically determined by Monte Carlo Simulation.

To clarify how the nuisance covariates included in the cortical thickness analysis contributed to language outcomes with and without cortical thickness measure in the model, the variables were further introduced into separate hierarchical linear regression with language outcomes as the dependent variables. Separate univariate analyses were performed to address group differences in mean cortical thickness in each identified cluster between patients and healthy subjects by controlling for the above demographic variables. Paired *t*-tests were conducted to compare white matter measures between the left and right hemispheres. Further, partial correlation analyses were performed to explore the relationships between cortical thickness in each cluster and mean FA values of the tracts connecting stroke lesions and cortical clusters by excluding the indicated demographic variables and lesion size. These analyses were performed using SPSS (version 22).

## Results

### Demographic details and language scores

The present study included 32 patients with subcortical stroke (24 infarcts and 8 hemorrhages) and 30 healthy control subjects. The demographic details and language scores of participants are shown in Table 1 and Supplementary Table 1. There were no significant differences in age, gender, educational level, or handedness between patients and healthy subjects (all *p* > 0.05). The mean time from stroke onset was 14.94 months (interquartile range = 7.8–35.7 months), and the mean lesion volume was 6.74 cm^3^ (interquartile range = 3.5–13.3 cm^3^). Among all patients, 26 suffered from aphasia and 6 patients were fully recovered. In aphasic patients, 25 (78%) were anomic aphasia. Significant differences were observed in the outcomes of naming/word finding [*F*(1, 56) = 18.31, *p* < 0.001], spontaneous speech [*F*(1, 56) = 8.87, *p* = 0.004] and auditory-verbal comprehension [*F*(1, 56) = 10.34, *p* = 0.002], but not repetition [*F*(1, 56) = 2.17, *p* = 0.146] in patients compared with healthy subjects when controlling for age, gender, educational level, and handedness.

**TABLE 1:** Demographic details and language performance in patients and healthy subjects.

	Patient group	Control group	Statistics	*P*-value
	(*n* = 32)	(*n* = 30)		
**Demographic variable**				
Age (years)	52.41 (13.23)	51.93 (12.98)	*t*(60) = 0.142	0.89
Gender (M/F)	21/11	19/11	χ^2^(1) = 0.04	0.85
Education (years)	10.81 (4.14)	11.70 (4.04)	*t*(60) = −0.85	0.40
Handedness (LQ)	84.66 (32.72)	85.33 (25.16)	*t*(62) = −0.09	0.93
Time post stroke (months)	24.72 (22.01)	–	–	–
Lesion size (cm^3^)	2.599 (2.14)	–	–	–
**Language assessment** *				
Naming/word-finding	8.29 (1.82)	9.66 (0.36)	*F*(1, 56) = 18.31^¶^	0.000
Auditory-verbal comprehension	9.47 (0.75)	9.92 (0.11)	*F*(1, 56) = 10.34^¶^	0.002
Repetition	9.68 (0.95)	9.98 (0.07)	*F*(1, 56) = 2.17^¶^	0.146
Spontaneous speech	18.41 (2.72)	19.91 (0.30)	*F*(1, 56) = 8.87^¶^	0.004

Parenthesis shows standard deviations. M, male; F, female; LQ, laterality quotient. n, number of subjects.

*Western Aphasia Battery-Revised subtests.

^¶^Factoring out age, gender, education level and handedness.

### Identification of critical areas related to language outcomes

We explored the potential associations of subcortical lesion location with different language scores by SVR-LSM. The results showed that no subcortical region in the left hemisphere in which damage relates to language scores was identified across patients (Data are not shown).

**FIGURE 2:**
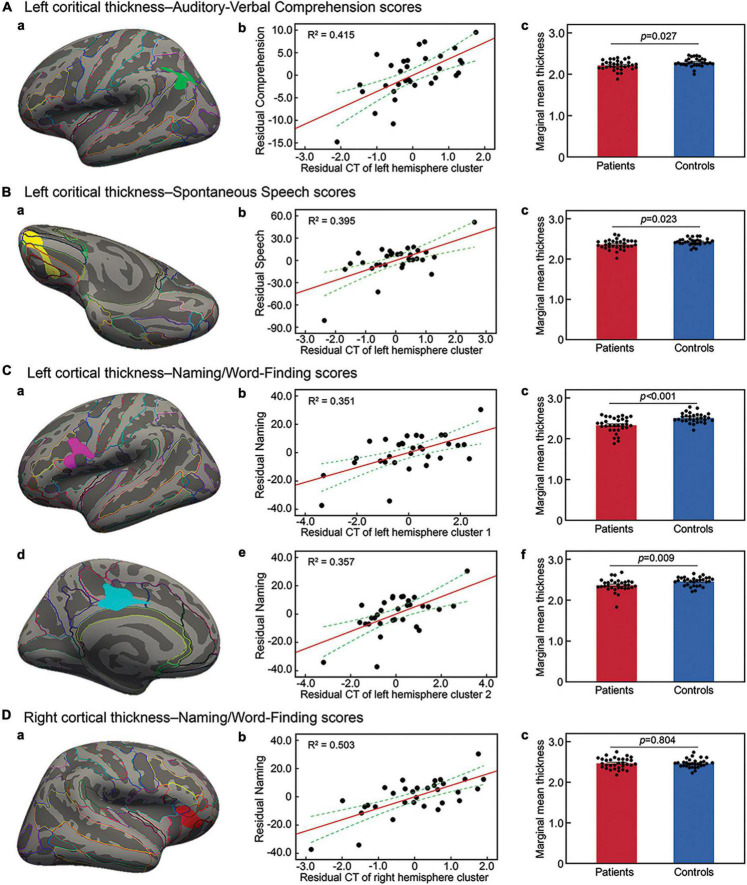
Cortical thickness in brain regions related to language outcomes. Surface representation of the significant clusters of cortical thickness related to the auditory-verbal comprehension score in the left inferior parietal cortex and superior temporal gyrus **(Aa)**, the spontaneous speech score in the left orbitofrontal gyrus **(Ba)**, and the naming/word finding score in the left inferior precentral gyrus **(Ca)**, left posterior cingulate gyrus **(Cd)**, and right orbitofrontal gyrus **(Da)**. All analyses were performed by controlling for demographic variables and lesion size, and corrected at a voxel-wise threshold of *P* < 0.005 and a cluster-wise level of *P* < 0.05, determined by Monte Carlo Simulation. **(Ab–Cb,Ce,Db)** Scatter plot showing partial regression using language scores as dependent variables and cortical thickness (CT) of clusters as independent factors by controlling for the above key variables (all *P* < 0.001). **(Ac–Cc,Cf,Dc)** Cortical thickness changes in each identified cluster in patients relative to controls factoring out demographic variables.

Next, separate regression analyses were performed to identify cortical thickness changes related to language scores by factoring out confounding variables. The correlation analysis showed education and lesion size significantly related to language outcomes (see Supplementary Table 2). Considering the possible impacts of age and time from stroke onset on cerebral volume (Nitkunan et al., 2011; Walhovd et al., 2011; Brodtmann et al., 2012; Takao et al., 2012), we included age, education level, time from stroke onset and lesion size as the key nuisances in the separate regression model. The results showed that cortical thickness in the left inferior parietal cortex and the posterior superior temporal gyrus was positively related to the auditory-verbal comprehension score (Figure 2Aa and Table 2). In contrast, cortical thickness in the left medial/lateral orbitofrontal gyrus was positively related to spontaneous speech outcome (Figure 2Ba and Table 2). As for naming ability, cortical thickness in the left inferior precentral gyrus, pars orbitalis, and left posterior cingulum was positively related to the naming/word finding score (Figures 2Ca,Cd and Table 2). In addition, cortical thickness in the right lateral orbitofrontal cortex, pars orbitalis, and pars triangularis was positively related to naming/word finding performance (Figure 2Da and Table 2). No negative relationships were found between cortical thickness and language performance.

**TABLE 2:** Clusters of cortical thickness related to language performance in patients.

Tests	Cluster size (mm^2^)	*P*_max_	Talairach coordinates (mm)			Anatomical regions
			*x*	*y*	*z*	
Multiple regression, Auditory-Verbal Comprehension as predictor (nuisance variables*)						
*LH*	803.25	0.009	–40.4	–66.1	35.3	Inferior Parietal Gyrus extending to
						Posterior Superior Temporal Gyrus
Multiple regression, Spontaneous Speech as predictor (nuisance variables*)						
*LH*	907.41	0.004	–6.2	54.3	–16.5	Medial Orbitofrontal Gyrus extending to
						Lateral Orbitofrontal Gyrus
Multiple regression, naming/word-finding as predictor (nuisance variables*)						
*LH*	659.04	0.029	–37.6	2.9	24.4	Inferior Precentral Gyrus extending to
						Pars Opercularis Gyrus
	641.38	0.032	–11.8	–18.6	37.4	Posterior Cingulate Gyrus
*RH*	576.24	0.045	41.5	30.5	–12.6	Lateral Orbitofrontal Gyrus extending to Pars Orbitalis and Triangularis

Significant clusters in each hemisphere are presented (corrected at vertex-wise P < 0.005 and cluster-wise P < 0.05). LH, left hemisphere; RH, right hemisphere.

*Nuisance variables include age, education level, time post stroke and lesion size.

To clarify the relationships between aphasia outcomes, cortical thickness measures, and confounding factors included in the above cortical thickness analyses, we next performed separate hierarchical regressions using the language scores as dependent variables. Age, education level, time from stroke onset and lesion size were entered first, followed by cortical thickness measures. When cortical thickness measure was excluded, the hierarchical regression analyses showed that only lesion size significantly predicted the abilities of auditory-verbal comprehension [*t*(27) = −4.11, *p* < 0.001], spontaneous speech [*t*(27) = −2.79, *p* = 0.010], and naming/word finding [*t*(26) = −3.56, *p* = 0.001]. When cortical thickness measure was added to the models, cortical thickness in the respective left hemisphere clusters was the only significant predictor for the corresponding language scores (auditory-verbal comprehension cluster: *R*^2^ change = 0.214, *p* < 0.001; spontaneous speech cluster: *R*^2^ change = 0.258, *p* < 0.001; naming/word finding cluster 1: *R*^2^ change = 0.171, *p* = 0.001 and cluster 2: *R*^2^ change = 0.065, *p* = 0.024). For naming, when cortical thickness measures in the clusters were all entered to the model, only the right hemisphere cluster was a significant predictor [*t*(24) = 2.20, *p* = 0.001]. Residual plots of the relationships between cortical thickness in the identified clusters and language performance scores are shown in Figures 2Ab–Cb,Ce,Db.

### Cortical thickness differences in patients compared with healthy subjects

Mean cortical thickness in the identified clusters was compared between aphasic patients and healthy subjects by factoring out age, gender, educational level, and handedness. Mean cortical thickness was significantly lower in patients than in healthy subjects in the left hemisphere clusters related to the outcomes of auditory-verbal comprehension [*F*(1, 56) = 5.13, *p* = 0.027; Figure 2Ac] and spontaneous speech [*F*(1, 56) = 5.47, *p* = 0.023; Figure 2Bc]. For naming/word finding, mean cortical thickness was significantly lower in patients than in healthy subjects in the left hemisphere clusters [cluster 1: *F*(1, 56) = 18.49, *p* < 0.001; cluster 2: *F*(1, 56) = 7.31, *p* = 0.009; Figures 2Cc,f]. In contrast, no significant difference was observed between groups in the mean cortical thickness in the right hemisphere cluster [*F*(1, 56) = 0.06, *p* = 0.804; Figure 2Dc].

### Correlation of connecting tract integrity with cortical thickness in patients

**FIGURE 3:**
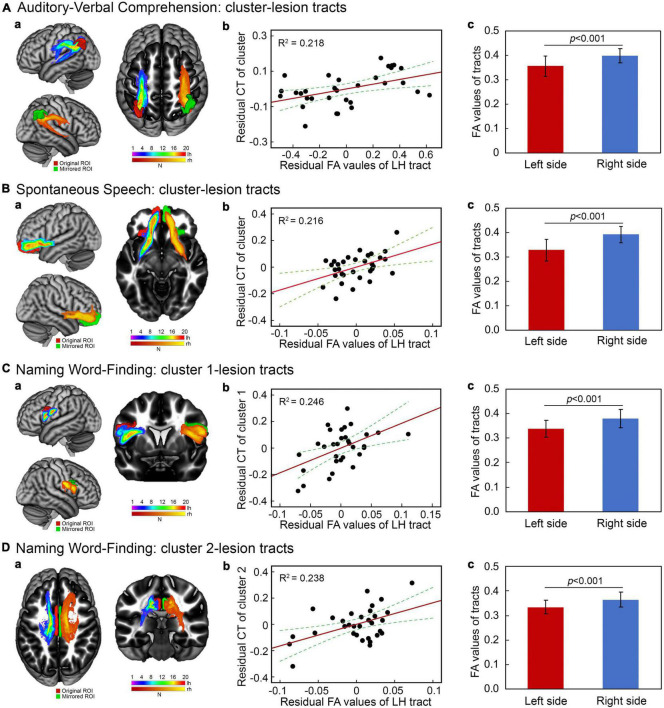
Integrity of white matter tracts related to cortical thickness in connecting brain regions. **(Aa–Da)** Reconstruction of fiber tracts connecting stroke lesions to cortical regions of interest (red) and the corresponding fiber tracts with mirrored stroke lesions and cortical regions related to language performance. Violet–red indicates the left hemisphere tracts and red–yellow indicates the mirrored tracts. All fiber tracking was thresholded at 1% of overall connectivity. **(Ab–Db)** Partial correlations between mean fractional anisotropy (FA) values of left fiber tracts and the corresponding cortical thickness measures when controlling for demographic variables and lesion size. **(Ac–Dc)** Paired comparisons of mean FA values of the left fiber tracts relative to their mirrored tracts (all *P* < 0.001).

White matter tracts connecting stroke lesion to the identified cortical regions and the mirrored homologous tracts were successfully reconstructed for all patients. The representative fiber tracts in a common space across patients are showed in Figures 3Aa–Da. To determine the potential correlates between cortical thickness in the identified regions with integrity of the reconstructed tracts, separate hierarchical regression analyses were performed with cortical thickness in each cluster as a dependent variable. The demographic variables, lesion size and FA values of connecting tracts were added to the models as independent factors. The results showed that mean FA values of the left hemisphere tracts were positively related to cortical thickness in the left hemisphere clusters related to the scores of auditory-verbal comprehension [*t*(24) = 2.59, *p* = 0.016], spontaneous speech [*t*(24) = 2.58, *p* = 0.017], and naming/word finding [cluster 1: *t*(24) = 2.80, *p* = 0.010; cluster 2: *t*(24) = 2.74, *p* = 0.011]. Residual plots of the relationships between cortical thickness in the identified clusters and language performance scores are shown in Figures 3Ab–Db. No significant relationships were found between cortical thickness in the right hemisphere cluster for naming/word finding and lesion size or the interhemispheric tracts connecting to lesions (both *p* > 0.20). In addition, mean FA value of each tract was significantly lower in the left hemisphere than in the right hemisphere homolog [*t*(31) = −5.49 ∼ –7.17, all *p* < 0.001] (Figures 3Ac–Dc).

### Comparisons of cortical thickness in subgroups with different perfusion status

**FIGURE 4:**
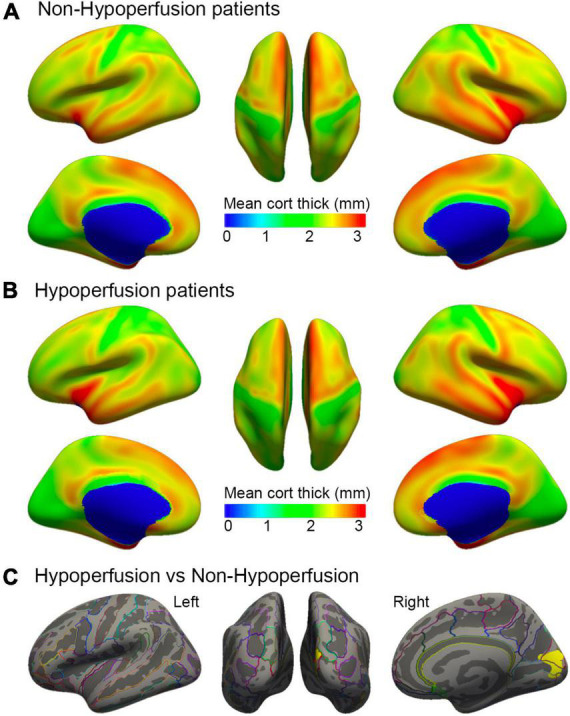
Comparisons of cortical thickness in patients with different perfusion status at stroke onset. Surface representation of mean cortical thickness for hemispheres in subgroups without hypoperfusion **(A)** and with hypoperfusion **(B)**. Color bar indicates cortical thickness changes in millimeter. **(C)** Representation of the statistically significant clusters showing a decline in cortical thickness in the right cuneus (cluster size = 1177.58 mm^2^; coordinates: *x* = 5.6, *y* = −67.7, *z* = 14.0; *P* = 0.0002, corrected for multiple comparisons), when controlling for demographic variables and lesion size.

To explore the impacts of perfusion status on cortical thickness, we further compared the differences in cortical thickness between patients without hypoperfusion (NHP) and with HP according to perfusion computer tomography or perfusion weighted images at stroke onset. Female patients were more [χ^2^(1) = 4.50, *p* = 0.03] and the lesion size was larger in the HP group than in the NHP group [*t*(30) = −2.12, *p* = 0.04]. No significant difference was detected for language performance (all *p* > 0.80) (see Supplementary Table 3). When controlling for demographic variables and lesion size, a reduction of cortical thickness was identified in the right cuneus regions (cluster size = 1177.58 mm^2^; coordinates: *x* = 5.6, *y* = −67.7, *z* = 14.0; *p* = 0.0002) in the HP group relative to the NHP group. There was no significant difference in the left hemisphere between the two subgroups (Figure 4). A *post hoc* analysis was performed to compare cortical thickness in each cluster identified as above to relate to language performance by controlling for key variables. The results showed that the mean cortical thickness of the individual clusters was not different between the two subgroups (all *p* > 0.11).

## Discussion

We found that cortical thickness changes in the left FTP regions were associated with language comprehension and production outcomes independent of inter-individual variability. Cortical thickness in the left FTP regions was lower in stroke patients with aphasia than in healthy subjects. Additionally, the cortical thinning was related to loss of microstructural integrity in fiber tracts connecting stroke lesions to cortical regions. Together, these findings suggest that remote secondary effects of subcortical stroke on the connecting cortical regions relate to language outcomes in chronic subcortical stroke.

### Associations of cortical thickness with subcortical aphasia outcomes

We found relationships between declined cortical thickness and subcortical aphasia outcomes by covarying key confounding variables. Specifically, auditory-verbal comprehension was predicted by cortical thickness in the left temporoparietal regions including the posterior temporal gyrus and inferior parietal cortex. It has been clarified that verbal comprehension relies on cortex considerably beyond the traditional location of Wernicke’s area in the posterior temporal gyrus (Turken and Dronkers, 2011; DeWitt and Rauschecker, 2012). Previous studies have suggested that the left inferior parietal cortex, especially the angular gyrus, is an essential area for speech comprehension as well as production (Mesulam, 1998; Vigneau et al., 2006). In contrast, Hickok and Poeppel (2007) proposed a model of the anatomy of language that excludes the angular gyrus from speech comprehension and production. However, it has been included by more recent studies (Lau et al., 2008; Hartwigsen et al., 2015). With respect to speech production, we found that declined thickness in the left orbitofrontal lobe negatively predicted speech outcome following chronic subcortical stroke. To support this, the PET study in healthy subjects has showed that activity in response to speech production to be localized in the left lateral and medial orbitofrontal cortex (Awad et al., 2007). Additionally, our results showed that reductions of thickness in the left inferior precentral gyrus/pars opercularis gyrus and posterior cingulate were strongly predictive of naming outcome. To support this, the left precentral cortex and inferior frontal gyrus are demonstrated to associate with action naming and the phonology component during object naming (Schwartz et al., 2012). Our results also accord with the observations in the previous study that increased activation could be observed within the left posterior cingulate area in healthy subjects during a picture identification task (Szaflarski et al., 2011).

Homotopic regions are traditionally thought to hinder language recovery by transcallosal disinhibition of the lesioned hemisphere (Belin et al., 1996; Blank et al., 2003; Naeser et al., 2005; Winhuisen et al., 2005). However, recent studies reveal beneficial effects of language homologs in the right hemisphere to post-stroke language recovery (Xing et al., 2016; Hope et al., 2017). Similarly, we found cortical thickness of the right lateral orbitofrontal cortex to be also related to naming outcome. This finding is further supported by a previous study in which damage to the right prefrontal cortex impaired the language ability of choosing appropriate words (Taubner et al., 1999). However, cortical thickness in the right hemisphere regions showed no considerable difference relative to controls and was neither related to lesion size nor integrity of connecting fiber tracts in the present study. This likely reflects premorbid inter-individual differences in the right hemisphere regions that protect from language deficits after left subcortical stroke. Patients with greater premorbid cortical thickness in the right hemisphere might have better recovery of language deficits after left subcortical stroke.

### Correlates of connecting tract integrity with cortical thickness in subcortical stroke

The current study demonstrated that there was a significant reduction of cortical thickness in the left hemisphere regions that related to language outcomes among patients as compared to the control subjects. These results might be indicative of secondary cortical atrophy in regions remote from stroke lesions as indicated in previous studies (Zhang et al., 2014). However, the exact mechanisms for structural impairment of the remote cortex remain unclear. It is assumed that fiber disconnection may account for this finding. By combining structural measurements with tractography, we found positive relationships between cortical thickness in the identified cortex and microstructural integrity of the connecting fiber tracts to subcortical lesions, but no significant correlation was seen the contralateral homologous tracts. Moreover, there was marked reductions of FA values in the tracts of lesional hemisphere relative to their contralateral homologs. These results might be reflective of secondary cortical effects of connecting fibers disruption after chronic subcortical stroke, as proposed by the diaschisis theory (Duering et al., 2012). Supporting this, previous studies have shown that focal cerebral infarction can induce progressive neuronal damage in the ipsilateral thalamus and substantia nigra remote from the lesion site (Nakane et al., 1992; Herve et al., 2005). Our results are also consistent with the findings of several longitudinal studies showing reduced thickness in the connecting cortical regions as a resultant of acute subcortical stroke (Cheng et al., 2015; Duering et al., 2015) or cortical remodeling in the bilateral hemisphere after basal ganglia infarction over several months (Liu et al., 2020). Additionally, retrograde neural degeneration of the retinal nerve fiber has been demonstrated among patients with occipital lobe stroke (Jindahra et al., 2012). Given the reconstructed tracts represent the anatomical connections between cortex and stroke lesions, these data implicate a possible link between subcortical aphasic stroke and ability to engage remote cortex in language processing secondary to fiber degeneration. Nevertheless, the casual effects of white matter disconnection on remote cortical atrophy cannot be determined from our results. The pathological mechanisms underlying the damage of connecting tracts are unclear. It might include retrograde or anterograde degeneration, dendrite shrinkage, and neuronal apoptosis because of loss of synaptic input (Siffrin et al., 2010).

Alternatively, acute subcortical aphasia might be resultant of concurrent cortical HP (Olsen et al., 1986; Wallesch et al., 1997). SPECT and perfusion weighted imaging measures revealed that focal lesion could cause remote cortical HP and hypometabolism (Carrera and Tononi, 2014). No significant differences in language scores were found between the two subgroups when controlling for key variables in the present study. In addition, the cortical thickness analysis showed no deference in cortical thickness of the left hemisphere in patients with HP relative to those with normal perfusion. This suggests that atrophy in the connected cortex was not driven by HP. However, cortical hypometabolism is also suggested in cases with subcortical aphasia (Halkar et al., 1997; de Boissezon et al., 2005; Sebastian et al., 2014). Additionally, specific cortical-subcortical circuits have been suggested to associate with language processing (Teichmann et al., 2015; Akinina et al., 2019; Jacquemot and Bachoud-Lévi, 2021). Thus, multiple neural mechanisms might contribute to aphasia outcomes after chronic subcortical stroke.

### Limitations

There are several limitations in the present study. First, this is a cross-sectional investigation that might preclude the empirical evidence of remote cortical atrophy mediated by neurodegeneration of the connecting fiber tracts. Further longitudinal study is expected to confirm the present findings by providing the dynamics of secondary neurodegeneration in remote cortical regions. Secondly, the DTI findings should be interpreted with caution. The extracellular volume effects of free water have been proposed to contaminate and invalidate DTI metrics (Pierpaoli et al., 1996; Pasternak et al., 2009). Additionally, it has been suggested that free-water correction would improve DTI-based tract reconstruction and tissue specificity (Metzler-Baddeley et al., 2012; Hoy et al., 2014; Guder et al., 2021). Considering the fact that atrophy and neuroinflammation can still be observed even several months after stroke, free-water correction will strengthen the reliability of DTI analysis especially in stroke patients. Thirdly, the number of patients with different perfusion status was relatively small in the subgroup analyses, which also restricted the generalization of the conclusions. Further study is need to confirm the present findings with a larger sample of populations. Fourthly, speech therapy experience across patients might drive recruitment of cerebral cortex capable of supporting language function, thereby affects the language outcome. However, the type and dose of speech therapy are not easily quantifiable as a confounding factor in the current regression analyses. Nevertheless, our results are not likely driven by a specific language therapy because the nature and quantity of speech therapy varies between individuals. Lastly, the lack of targeted therapeutic intervention, such as the restoration of cerebral perfusion, was a limitation of the study. Direct intervention of cerebral flow should permit inferences on the HP effects on cortical thickness changes or language outcomes as previously reported (Hillis et al., 2002). Notably, our results were obtained by using FreeSurfer software that could detect changes in cortical thickness at the submillimeter scale. Additionally, the present study demonstrates the feasibility of investigating the neural base for subcortical aphasia with a combination of surface-based thickness analysis with connecting fiber tracking.

## Conclusion

Our data provide direct evidence that cortical thickness in the left FTP regions is correlated with language outcomes in patients with chronic subcortical stroke and aphasia. The loss of microstructural integrity in connecting fiber tracts to stroke lesions might serve as the underlying mechanism for remote cortical atrophy in subcortical aphasia. These findings will improve the understanding of cerebral reorganization and language recovery after chronic subcortical stroke.

## Data availability statement

The raw data supporting the conclusions of this article will be made available by the authors, without undue reservation.

## Ethics statement

The studies involving human participants were reviewed and approved by Institutional Review Board of the First Affiliated Hospital of Sun Yat-sen University. The patients/participants provided their written informed consent to participate in this study. Written informed consent was obtained from the individual(s) for the publication of any potentially identifiable images or data included in this article.

## Author contributions

HT was responsible for data collection, analysis, and manuscript drafting. SF, XN, PX, and ZL collected the data. SX designed the work, analyzed the data, and drafted and revised the manuscript. All authors gave their final approval of the version to be published and agreed to be accountable for all aspects of the work.

## Footnotes

1
https://www.nitrc.org/projects/mricron

2
http://www.neuro.uni-jena.de/cat/

3
http://surfer.nmr.mgh.harvard.edu

4
http://www.fmrib.ox.ac.uk/fsl/

## References

[B1] Akinina Y. Dragoy O. Ivanova M. V. Iskra E. V. Soloukhina O. A. Petryshevsky A. G. (2019). Grey and white matter substrates of action naming. *Neuropsychologia* 131 249–265. 10.1016/j.neuropsychologia.2019.05.015 31129278 PMC6650369

[B2] Awad M. Warren J. E. Scott S. K. Turkheimer F. E. Wise R. J. (2007). A common system for the comprehension and production of narrative speech. *J. Neurosci.* 27 11455–11464. 10.1523/JNEUROSCI.5257-06.2007 17959788 PMC6673222

[B3] Behrens T. E. Berg H. J. Jbabdi S. Rushworth M. F. Woolrich M. W. (2007). Probabilistic diffusion tractography with multiple fibre orientations: What can we gain? *Neuroimage* 34 144–155. 10.1016/j.neuroimage.2006.09.018 17070705 PMC7116582

[B4] Belin P. Van Eeckhout P. Zilbovicius M. Remy P. François C. Guillaume S. (1996). Recovery from nonfluent aphasia after melodic intonation therapy: A PET study. *Neurology* 47 1504–1511. 10.1212/wnl.47.6.1504 8960735

[B5] Blank S. C. Bird H. Turkheimer F. Wise R. J. (2003). Speech production after stroke: The role of the right pars opercularis. *Ann. Neurol.* 54 310–320. 10.1002/ana.10656 12953263

[B6] Brodtmann A. Pardoe H. Li Q. Lichter R. Ostergaard L. Cumming T. (2012). Changes in regional brain volume three months after stroke. *J. Neurol. Sci.* 322 122–128. 10.1016/j.jns.2012.07.019 22858417

[B7] Carrera E. Tononi G. (2014). Diaschisis: Past, present, future. *Brain* 137 2408–2422. 10.1093/brain/awu101 24871646

[B8] Cheng B. Dietzmann P. Schulz R. Boenstrup M. Krawinkel L. Fiehler J. (2020). Cortical atrophy and transcallosal diaschisis following isolated subcortical stroke. *J. Cereb. Blood Flow Metab.* 40 611–621. 10.1177/0271678X19831583 30782059 PMC7026841

[B9] Cheng B. Schulz R. Bonstrup M. Hummel F. C. Sedlacik J. Fiehler J. (2015). Structural plasticity of remote cortical brain regions is determined by connectivity to the primary lesion in subcortical stroke. *J. Cereb. Blood Flow Metab.* 35 1507–1514. 10.1038/jcbfm.2015.74 25920957 PMC4640340

[B10] Choi J. Y. Lee K. H. Na D. L. Byun H. S. Lee S. J. Kim H. (2007). Subcortical aphasia after striatocapsular infarction: Quantitative analysis of brain perfusion SPECT using statistical parametric mapping and a statistical probabilistic anatomic map. *J. Nucl. Med.* 48 194–200. 17268014

[B11] Dang C. Liu G. Xing S. Xie C. Peng K. Li C. (2013). Longitudinal cortical volume changes correlate with motor recovery in patients after acute local subcortical infarction. *Stroke* 44 2795–2801. 10.1161/STROKEAHA.113.000971 23929747

[B12] de Boissezon X. Demonet J. F. Puel M. Marie N. Raboyeau G. Albucher J. F. (2005). Subcortical aphasia: A longitudinal PET study. *Stroke* 36 1467–1473. 10.1161/01.STR.0000169947.08972.4f 15933252

[B13] DeMarco A. T. Turkeltaub P. E. (2018). A multivariate lesion symptom mapping toolbox and examination of lesion-volume biases and correction methods in lesion-symptom mapping. *Hum. Brain Mapp.* 39 4169–4182. 10.1002/hbm.24289 29972618 PMC6647024

[B14] DeWitt I. Rauschecker J. P. (2012). Phoneme and word recognition in the auditory ventral stream. *Proc. Natl. Acad. Sci. U.S.A.* 109 E505–E514. 10.1073/pnas.1113427109 22308358 PMC3286918

[B15] Dronkers N. F. Ivanova M. V. Baldo J. V. (2017). What Do Language Disorders Reveal about Brain-Language Relationships? From Classic Models to Network Approaches. *J. Int. Neuropsychol. Soc.* 23 741–754. 10.1017/S1355617717001126 29198286 PMC6606454

[B16] Duering M. Righart R. Csanadi E. Jouvent E. Hervé D. Chabriat H. (2012). Incident subcortical infarcts induce focal thinning in connected cortical regions. *Neurology* 79 2025–2028. 10.1212/WNL.0b013e3182749f39 23054230

[B17] Duering M. Righart R. Wollenweber F. A. Zietemann V. Gesierich B. Dichgans M. (2015). Acute infarcts cause focal thinning in remote cortex via degeneration of connecting fiber tracts. *Neurology* 84 1685–1692. 10.1212/wnl.0000000000001502 25809303 PMC4409580

[B18] Fischl B. Salat D. H. van der Kouwe A. J. Makris N. Segonne F. Quinn B. T. (2004). Sequence-independent segmentation of magnetic resonance images. *Neuroimage* 23 S69–S84. 10.1016/j.neuroimage.2004.07.016 15501102

[B19] Guder S. Pasternak O. Gerloff C. Schulz R. (2021). Strengthened structure-function relationships of the corticospinal tract by free water correction after stroke. *Brain Commun.* 3:fcab034. 10.1093/braincomms/fcab034 33959708 PMC8088790

[B20] Halkar R. K. Sisterhen C. Ammons J. Galt J. R. Alazraki N. P. (1997). Tc-99m ECD SPECT imaging in aphasia caused by subcortical infarct. *Clin. Nucl. Med.* 22 850–851. 10.1097/00003072-199712000-00010 9408649

[B21] Hartwigsen G. Golombek T. Obleser J. (2015). Repetitive transcranial magnetic stimulation over left angular gyrus modulates the predictability gain in degraded speech comprehension. *Cortex* 68 100–110. 10.1016/j.cortex.2014.08.027 25444577

[B22] Herve D. Molko N. Pappata S. Buffon F. LeBihan D. Bousser M. G. (2005). Longitudinal thalamic diffusion changes after middle cerebral artery infarcts. *J. Neurol. Neurosurg. Psychiatry* 76 200–205. 10.1136/jnnp.2004.041012 15654032 PMC1739509

[B23] Hickok G. Poeppel D. (2007). The cortical organization of speech processing. *Nat. Rev. Neurosci.* 8 393–402. 10.1038/nrn2113 17431404

[B24] Hillis A. E. Wityk R. J. Barker P. B. Beauchamp N. J. Gailloud P. Murphy K. (2002). Subcortical aphasia and neglect in acute stroke: The role of cortical hypoperfusion. *Brain* 125 1094–1104. 10.1093/brain/awf113 11960898

[B25] Hope T. M. H. Leff A. P. Prejawa S. Bruce R. Haigh Z. Lim L. (2017). Right hemisphere structural adaptation and changing language skills years after left hemisphere stroke. *Brain* 140 1718–1728. 10.1093/brain/awx086 28444235 PMC5445256

[B26] Hoy A. R. Koay C. G. Kecskemeti S. R. Alexander A. L. (2014). Optimization of a free water elimination two-compartment model for diffusion tensor imaging. *Neuroimage* 103 323–333. 10.1016/j.neuroimage.2014.09.053 25271843 PMC4312191

[B27] Jacquemot C. Bachoud-Lévi A. C. (2021). Striatum and language processing: Where do we stand? *Cognition* 213:104785. 10.1016/j.cognition.2021.104785 34059317

[B28] Jindahra P. Petrie A. Plant G. T. (2012). The time course of retrograde trans-synaptic degeneration following occipital lobe damage in humans. *Brain* 135 534–541. 10.1093/brain/awr324 22300877

[B29] Kuljic-Obradovic D. C. (2003). Subcortical aphasia: Three different language disorder syndromes? *Eur. J. Neurol.* 10 445–448. 10.1046/j.1468-1331.2003.00604.x 12823499

[B30] Lau E. F. Phillips C. Poeppel D. (2008). A cortical network for semantics: (de)constructing the N400. *Nat. Rev. Neurosci.* 9 920–933. 10.1038/nrn2532 19020511

[B31] Liu H. Peng X. Dahmani L. Wang H. Zhang M. Shan Y. (2020). Patterns of motor recovery and structural neuroplasticity after basal ganglia infarcts. *Neurology* 95:e1174–e1187. 10.1212/WNL.0000000000010149 32586896 PMC7538227

[B32] Lotan E. Tavor I. Barazany D. Ben-Amitay S. Hoffmann C. Tsarfaty G. (2019). Selective atrophy of the connected deepest cortical layers following small subcortical infarct. *Neurology* 92 e567–e575. 10.1212/wnl.0000000000006884 30635479

[B33] Mesulam M. M. (1998). From sensation to cognition. *Brain* 121 1013–1052. 10.1093/brain/121.6.1013 9648540

[B34] Metzler-Baddeley C. O’Sullivan M. J. Bells S. Pasternak O. Jones D. K. (2012). How and how not to correct for CSF-contamination in diffusion MRI. *Neuroimage* 59 1394–1403. 10.1016/j.neuroimage.2011.08.043 21924365

[B35] Nachev P. Coulthard E. Jager H. R. Kennard C. Husain M. (2008). Enantiomorphic normalization of focally lesioned brains. *Neuroimage* 39 1215–1226. 10.1016/j.neuroimage.2007.10.002 18023365 PMC2658465

[B36] Naeser M. A. Alexander M. P. Helm-Estabrooks N. Levine H. L. Laughlin S. A. Geschwind N. (1982). Aphasia with predominantly subcortical lesion sites: Description of three capsular/putaminal aphasia syndromes. *Arch. Neurol.* 39 2–14. 10.1001/archneur.1982.00510130004002 6976780

[B37] Naeser M. A. Martin P. I. Nicholas M. Baker E. H. Seekins H. Kobayashi M. (2005). Improved picture naming in chronic aphasia after TMS to part of right Broca’s area: An open-protocol study. *Brain Lang.* 93 95–105. 10.1016/j.bandl.2004.08.004 15766771

[B38] Nakane M. Teraoka A. Asato R. Tamura A. (1992). Degeneration of the ipsilateral substantia nigra following cerebral infarction in the striatum. *Stroke* 23 328–332. 10.1161/01.str.23.3.328 1542891

[B39] Nitkunan A. Lanfranconi S. Charlton R. A. Barrick T. R. Markus H. S. (2011). Brain atrophy and cerebral small vessel disease: A prospective follow-up study. *Stroke* 42 133–138. 10.1161/strokeaha.110.594267 21148440

[B40] Noh J. S. Lee S. Na Y. Cho M. Hwang Y. M. Tae W.-S. (2021). Integrity of arcuate fasciculus is a good predictor of language impairment after subcortical stroke. *J. Neurolinguist.* 58:100968. 10.1016/j.jneuroling.2020.100968

[B41] Okuda B. Tanaka H. Tachibana H. Kawabata K. Sugita M. (1994). Cerebral blood flow in subcortical global aphasia. Perisylvian cortical hypoperfusion as a crucial role. *Stroke* 25 1495–1499. 10.1161/01.str.25.7.1495 8023368

[B42] Olsen T. S. Bruhn P. Oberg R. G. (1986). Cortical hypoperfusion as a possible cause of ‘subcortical aphasia’. *Brain* 109 393–410. 10.1093/brain/109.3.393 2424544

[B43] Pasternak O. Sochen N. Gur Y. Intrator N. Assaf Y. (2009). Free water elimination and mapping from diffusion MRI. *Magn. Reson. Med.* 62 717–730. 10.1002/mrm.22055 19623619

[B44] Peñaloza C. Rodríguez-Fornells A. Rubio F. De Miquel M. A. Juncadella M. (2014). Language recovery and evidence of residual deficits after nonthalamic subcortical stroke: A 1 year follow-up study. *J. Neurolinguist.* 32 16–30. 10.1016/j.jneuroling.2014.08.001

[B45] Perani D. Vallar G. Cappa S. Messa C. Fazio F. (1987). Aphasia and neglect after subcortical stroke. A clinical/cerebral perfusion correlation study. *Brain* 110 1211–1229. 10.1093/brain/110.5.1211 3499949

[B46] Pierpaoli C. Jezzard P. Basser P. J. Barnett A. Di Chiro G. (1996). Diffusion tensor MR imaging of the human brain. *Radiology* 201 637–648. 10.1148/radiology.201.3.8939209 8939209

[B47] Schwartz M. F. Faseyitan O. Kim J. Coslett H. B. (2012). The dorsal stream contribution to phonological retrieval in object naming. *Brain* 135 3799–3814. 10.1093/brain/aws300 23171662 PMC3525060

[B48] Sebastian R. Schein M. G. Davis C. Gomez Y. Newhart M. Oishi K. (2014). Aphasia or Neglect after Thalamic Stroke: The Various Ways They may be Related to Cortical Hypoperfusion. *Front. Neurol.* 5:231. 10.3389/fneur.2014.00231 25477859 PMC4237053

[B49] Shewan C. M. Kertesz A. (1980). Reliability and validity characteristics of the Western Aphasia Battery (WAB). *J. Speech Hear. Disord.* 45 308–324. 10.1044/jshd.4503.308 7412225

[B50] Siffrin V. Vogt J. Radbruch H. Nitsch R. Zipp F. (2010). Multiple sclerosis - candidate mechanisms underlying CNS atrophy. *Trends Neurosci.* 33 202–210. 10.1016/j.tins.2010.01.002 20153532

[B51] Smith S. M. Jenkinson M. Woolrich M. W. Beckmann C. F. Behrens T. E. Johansen-Berg H. (2004). Advances in functional and structural MR image analysis and implementation as FSL. *Neuroimage* 23 S208–S219. 10.1016/j.neuroimage.2004.07.051 15501092

[B52] Szaflarski J. P. Eaton K. Ball A. L. Banks C. Vannest J. Allendorfer J. B. (2011). Poststroke aphasia recovery assessed with functional magnetic resonance imaging and a picture identification task. *J. Stroke Cerebrovasc. Dis.* 20 336–345. 10.1016/j.jstrokecerebrovasdis.2010.02.003 20719532 PMC2990790

[B53] Takao H. Hayashi N. Ohtomo K. (2012). A longitudinal study of brain volume changes in normal aging. *Eur. J. Radiol.* 81 2801–2804. 10.1016/j.ejrad.2011.10.011 22104089

[B54] Taubner R. W. Raymer A. M. Heilman K. M. (1999). Frontal-opercular aphasia. *Brain Lang.* 70 240–261. 10.1006/brln.1999.2157 10550229

[B55] Teichmann M. Rosso C. Martini J. B. Bloch I. Brugieres P. Duffau H. (2015). A cortical-subcortical syntax pathway linking Broca’s area and the striatum. *Hum. Brain Mapp.* 36 2270–2283. 10.1002/hbm.22769 25682763 PMC6869141

[B56] Turken A. U. Dronkers N. F. (2011). The neural architecture of the language comprehension network: Converging evidence from lesion and connectivity analyses. *Front. Syst. Neurosci.* 5:1. 10.3389/fnsys.2011.00001 21347218 PMC3039157

[B57] Vallar G. Perani D. Cappa S. F. Messa C. Lenzi G. L. Fazio F. (1988). Recovery from aphasia and neglect after subcortical stroke: Neuropsychological and cerebral perfusion study. *J. Neurol. Neurosurg. Psychiatry* 51 1269–1276. 10.1136/jnnp.51.10.1269 2465386 PMC1032915

[B58] Vigneau M. Beaucousin V. Herve P. Y. Duffau H. Crivello F. Houde O. (2006). Meta-analyzing left hemisphere language areas: Phonology, semantics, and sentence processing. *Neuroimage* 30 1414–1432. 10.1016/j.neuroimage.2005.11.002 16413796

[B59] Walhovd K. B. Westlye L. T. Amlien I. Espeseth T. Reinvang I. Raz N. (2011). Consistent neuroanatomical age-related volume differences across multiple samples. *Neurobiol. Aging* 32 916–932. 10.1016/j.neurobiolaging.2009.05.013 19570593 PMC4040218

[B60] Wallesch C. W. Johannsen-Horbach H. Bartels C. Herrmann M. (1997). Mechanisms of and misconceptions about subcortical aphasia. *Brain Lang.* 58 403–409. 10.1006/brln.1997.1805 9222519

[B61] Winhuisen L. Thiel A. Schumacher B. Kessler J. Rudolf J. Haupt W. F. (2005). Role of the contralateral inferior frontal gyrus in recovery of language function in poststroke aphasia: A combined repetitive transcranial magnetic stimulation and positron emission tomography study. *Stroke* 36 1759–1763. 10.1161/01.STR.0000174487.81126.ef 16020770

[B62] Worrall B. B. Farace E. Hillis A. E. Hutson R. K. Wityk R. Saver J. L. (2001). Correlation of aphasia and/or neglect with cortical infarction in a subpopulation of RANTTAS. *Cerebrovasc. Dis.* 11 257–264. 11306777 10.1159/000047648

[B63] Xing S. Lacey E. H. Skipper-Kallal L. M. Jiang X. Harris-Love M. L. Zeng J. (2016). Right hemisphere grey matter structure and language outcomes in chronic left hemisphere stroke. *Brain* 139 227–241. 10.1093/brain/awv323 26521078 PMC4990653

[B64] Xing S. Mandal A. Lacey E. H. Skipper-Kallal L. M. Zeng J. Turkeltaub P. E. (2018). Behavioral Effects of Chronic Gray and White Matter Stroke Lesions in a Functionally Defined Connectome for Naming. *Neurorehabil. Neural. Repair* 32 613–623. 10.1177/1545968318780351 29890878 PMC6051910

[B65] Zhang B. Chang J. Park J. Tan Z. Tang L. Lyu T. (2021). Uncinate fasciculus and its cortical terminals in aphasia after subcortical stroke: A multi-modal MRI study. *Neuroimage Clin.* 30:102597. 10.1016/j.nicl.2021.102597 33684729 PMC7941046

[B66] Zhang J. Meng L. Qin W. Liu N. Shi F. D. Yu C. (2014). Structural damage and functional reorganization in ipsilesional m1 in well-recovered patients with subcortical stroke. *Stroke* 45 788–793. 10.1161/STROKEAHA.113.003425 24496396

