# Supplementary material for: Remote cortical atrophy and language outcomes after chronic left subcortical stroke with aphasia
Source: Front Neurosci. 2022 Aug 3;16:853169. doi: 10.3389/fnins.2022.853169 (PMC9381815; doi:10.3389/fnins.2022.853169)
Supplement: Supplementary file 1 [file Table_1.docx]

**Supplementary Table 1. Patient demographic details and language scores.**

| **Patients** | **Age (years)** | **Gender** | **Education (years)** | **Handedness (LQ)** | **Time post stroke (months)** | **Lesion size (cm^3^)** | **Stroke Type** | **Aphasia Type** | **AQ** | **Speech** | **Com** | **Naming** | **Repet** |
| --- | --- | --- | --- | --- | --- | --- | --- | --- | --- | --- | --- | --- | --- |
| **1** | 76 | M | 16.00 | 100.00 | 59.13 | 13.42 | Ischemic | Anomic | 89.80 | 18.00 | 9.30 | 7.80 | 9.80 |
| **2** | 56 | M | 15.00 | 100.00 | 44.97 | 17.71 | Hemorrhagic | Anomic | 88.70 | 18.00 | 9.15 | 7.30 | 9.90 |
| **3** | 51 | M | 7.00 | 100.00 | 51.83 | 4.29 | Hemorrhagic | Anomic | 92.20 | 19.00 | 8.90 | 8.50 | 9.70 |
| **4** | 61 | M | 9.00 | 100.00 | 6.07 | 5.62 | Hemorrhagic | Anomic | 96.00 | 19.00 | 10.00 | 9.00 | 10.00 |
| **5** | 49 | M | 18.00 | 100.00 | 6.70 | 3.79 | Hemorrhagic | Recovery | 96.80 | 19.00 | 10.00 | 9.60 | 9.80 |
| **6** | 41 | M | 12.00 | 92.00 | 26.83 | 4.72 | Hemorrhagic | Anomic | 97.10 | 20.00 | 9.65 | 9.00 | 9.90 |
| **7** | 29 | F | 16.00 | 82.00 | 36.10 | 13.69 | Ischemic | Anomic | 98.60 | 20.00 | 10.00 | 9.40 | 9.90 |
| **8** | 65 | F | 9.00 | 91.00 | 21.17 | 5.25 | Ischemic | Anomic | 95.20 | 19.00 | 9.70 | 9.30 | 9.60 |
| **9** | 51 | M | 8.00 | 100.00 | 6.07 | 1.67 | Ischemic | Anomic | 96.00 | 19.00 | 10.00 | 9.00 | 10.00 |
| **10** | 53 | M | 12.00 | 92.00 | 26.67 | 1.47 | Ischemic | Anomic | 98.00 | 20.00 | 10.00 | 9.00 | 10.00 |
| **11** | 53 | M | 8.00 | 100.00 | 9.07 | 8.07 | Ischemic | Anomic | 93.50 | 19.00 | 9.25 | 8.50 | 10.00 |
| **12** | 39 | M | 8.00 | 100.00 | 6.43 | 8.67 | Ischemic | Anomic | 87.30 | 18.00 | 9.25 | 6.40 | 10.00 |
| **13** | 65 | F | 6.00 | 100.00 | 29.83 | .51 | Ischemic | Anomic | 91.50 | 19.00 | 9.35 | 7.50 | 9.90 |
| **14** | 40 | M | 15.00 | 33.00 | 14.07 | 4.95 | Ischemic | Recovery | 100.00 | 20.00 | 10.00 | 10.00 | 10.00 |
| **15** | 45 | F | 5.00 | 100.00 | 9.83 | 29.66 | Ischemic | Anomic | 90.00 | 19.00 | 8.20 | 7.80 | 10.00 |
| **16** | 57 | F | 5.00 | 100.00 | 9.57 | 18.30 | Ischemic | Broca's | 42.90 | 7.00 | 6.95 | 2.20 | 5.30 |
| **17** | 32 | M | 9.00 | 42.00 | 66.80 | 19.58 | Ischemic | Anomic | 97.80 | 20.00 | 9.80 | 9.10 | 10.00 |
| **18** | 30 | M | 9.00 | 20.00 | 6.90 | 10.26 | Ischemic | Anomic | 90.40 | 18.00 | 9.50 | 7.70 | 10.00 |
| **19** | 45 | M | 19.00 | 92.00 | 49.67 | .34 | Ischemic | Recovery | 99.60 | 20.00 | 10.00 | 9.80 | 10.00 |
| **20** | 69 | M | 6.00 | 90.00 | 34.40 | 14.86 | Ischemic | Anomic | 93.50 | 19.00 | 9.95 | 8.00 | 9.80 |
| **21** | 76 | M | 17.00 | 91.00 | 20.83 | 11.07 | Ischemic | Anomic | 95.30 | 19.00 | 9.85 | 8.80 | 10.00 |
| **22** | 51 | M | 12.00 | 92.00 | 18.57 | 2.91 | Ischemic | Recovery | 99.00 | 20.00 | 10.00 | 9.50 | 10.00 |
| **23** | 56 | M | 8.00 | -58.00 | 8.27 | 3.77 | Ischemic | Anomic | 90.00 | 18.00 | 9.40 | 7.60 | 10.00 |
| **24** | 40 | M | 8.00 | 91.00 | 15.80 | 1.96 | Hemorrhagic | Anomic | 92.90 | 19.00 | 9.55 | 8.70 | 9.20 |
| **25** | 54 | M | 15.00 | 83.00 | 13.23 | 10.51 | Ischemic | Anomic | 94.30 | 19.00 | 9.55 | 8.70 | 9.90 |
| **26** | 63 | F | 8.00 | 100.00 | 8.27 | 24.24 | Ischemic | Anomic | 52.70 | 10.00 | 7.15 | 2.00 | 7.20 |
| **27** | 31 | F | 16.00 | 100.00 | 70.90 | 4.36 | Ischemic | Recovery | 99.20 | 20.00 | 9.70 | 9.90 | 10.00 |
| **28** | 67 | F | 6.00 | 90.00 | 83.07 | 8.60 | Hemorrhagic | Anomic | 97.10 | 20.00 | 9.75 | 8.80 | 10.00 |
| **29** | 60 | F | 11.00 | 100.00 | 9.67 | 12.88 | Ischemic | Anomic | 92.50 | 18.00 | 9.75 | 8.50 | 10.00 |
| **30** | 49 | M | 9.00 | 100.00 | 6.03 | 3.45 | Ischemic | Anomic | 96.20 | 19.00 | 9.90 | 9.20 | 10.00 |
| **31** | 48 | F | 15.00 | 100.00 | 6.70 | 7.85 | Ischemic | Recovery | 96.40 | 19.00 | 9.80 | 9.40 | 10.00 |
| **32** | 75 | F | 9.00 | 86.00 | 7.60 | 1.93 | Ischemic | Anomic | 94.00 | 18.00 | 9.80 | 9.20 | 10.00 |

M = male; F = female; LQ = laterality quotient, a handedness measure [range = -100% (strongly left-handed) to 100% (strongly right-handed)]; AQ = Aphasia Quotient; Naming = Naming/Word Finding; Com = Auditory-Verbal Comprehension; Repet = Repetition; Speech = Spontaneous Speech; TSA = transcortical sensory aphasia.
