# Supplementary material for: Remote cortical atrophy and language outcomes after chronic left subcortical stroke with aphasia
Source: Front Neurosci. 2022 Aug 3;16:853169. doi: 10.3389/fnins.2022.853169 (PMC9381815; doi:10.3389/fnins.2022.853169)
Supplement: Supplementary file 2 [file Table_2.docx]

**Supplementary Table 2.** **Correlation coefficients between demographic variables, lesion size, time form stroke onset and language scores across patients.**

|  | Auditory-Verbal Comprehension | | Spontaneous Speech | | Naming/  Word-Finding | | Repetition | |
| --- | --- | --- | --- | --- | --- | --- | --- | --- |
| Age | | -0.118 | | -0.231 | | -0.216 | | -0.133 |
| Gender | | -0.322 | | -0.331 | | -0.263 | | -0.327 |
| Education level | | 0.434^*^ | | 0.328 | | 0.443^*^ | | 0.303 |
| Time from onset | | 0.147 | | 0.285 | | 0.212 | | 0.170 |
| Handedness | | -0.116 | | -0.081 | | -0.054 | | -0.144 |
| Lesion size | | -0.622^**^ | | -0.458^**^ | | -0.557^**^ | | -0.384^*^ |

^*^*P* < 0.05; ^**^*P* < 0.01.
