# Supplementary material for: Remote cortical atrophy and language outcomes after chronic left subcortical stroke with aphasia
Source: Front Neurosci. 2022 Aug 3;16:853169. doi: 10.3389/fnins.2022.853169 (PMC9381815; doi:10.3389/fnins.2022.853169)
Supplement: Supplementary file 3 [file Table_3.docx]

**Supplementary Table 3. Demographic details and language outcomes in patients with different perfusion status at stroke onset.**

|  | **NHP group** | **HP group** | **Statistics** | | ***P*-value** |
| --- | --- | --- | --- | --- | --- |
|  | **(*n* = 17)** | **(*n* = 15)** |  | |  |
| **Demographic variable** | | |  | |  |
| Age (years) | 54.35 (11.85) | 50.20 (14.74) | *t*(30) = 0.88 | | 0.38 |
| Gender (M/F) | 14/3 | 7/8 | *χ*^2^(1) = 4.50 | | 0.03 |
| Education (years) | 11.00 (4.23) | 10.60 (4.10) | *t*(30) = 0.27 | | 0.79 |
| Handedness (R/L) | 16/1 | 14/1 | *χ*^2^(1) = 0.52 | | 0.47 |
| Time post stroke (months) | 29.47 (21.94) | 19.34 (21.54) | *t*(30) = 1.31 | | 0.20 |
| Lesion size (cm^3^) | 6.35 (5.31) | 11.50 (8.25) | *t*(30) = -2.12 | | 0.04 |
| **Language assessment**^*^ | | |  |  |  |
| Naming/Word-Finding | 8.66 (0.75) | 7.87 (2.52) | *F*(1,24) = 0.006^¶^ | | 0.94 |
| Auditory-Verbal Comprehension | 9.67 (0.35) | 9.25 (1.00) | *F*(1,24) = 0.004^¶^ | | 0.95 |
| Repetition | 9.85 (0.21) | 9.49 (1.37) | *F*(1,24) = 0.005^¶^ | | 0.95 |
| Spontaneous Speech | 19.12 (0.70) | 17.60 (3.81) | *F*(1,24) = 0.051^¶^ | | 0.82 |

Standard deviations are presented in parenthesis. NHP, non-hypoperfusion; HP, hypoperfusion; M, male; F, female. *n*, number of subjects.

^*^Western Aphasia Battery-Revised subtest. ^¶^Factoring out age, gender, years of education, handedness, time from stroke and lesion size.
