# Supplementary material for: Remote cortical atrophy and language outcomes after chronic left subcortical stroke with aphasia
Source: Front Neurosci. 2022 Aug 3;16:853169. doi: 10.3389/fnins.2022.853169 (PMC9381815; doi:10.3389/fnins.2022.853169)
Supplement: Supplementary file 4 [file Image_1.pdf]

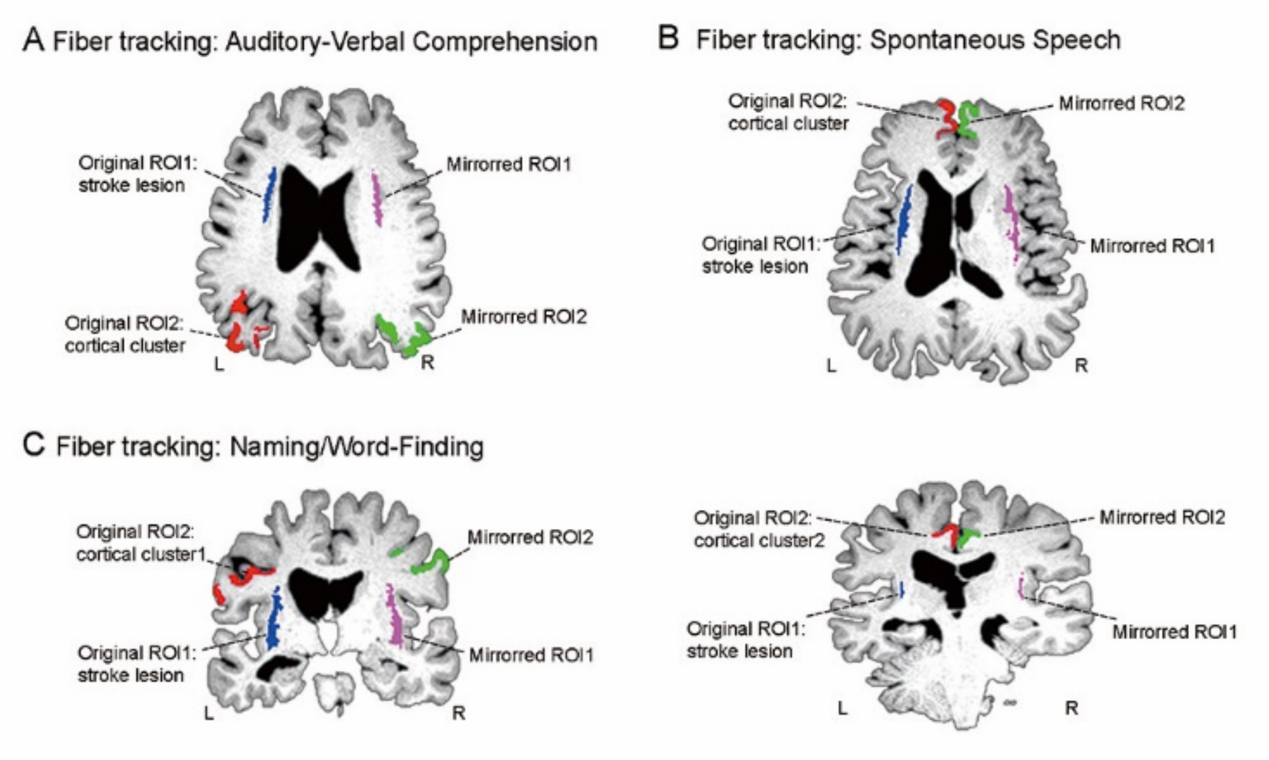

Supplementary Figure 1. Representative region of interests (ROIs) for fiber tracking in native space from one patient. (A-C) Tractography was performed between the stroke seed (original ROI1) and the cortical target that related to the respective language outcomes in the native space (original ROI2). The homologous tracts in the contralateral hemisphere were tracked between the control region stroke lesion (mirrored ROI1) and the control cortical target (mirrored ROI2).
